# Identification of Reference Genes for Quantitative Real Time PCR Assays in Aortic Tissue of Syrian Hamsters with Bicuspid Aortic Valve

**DOI:** 10.1371/journal.pone.0164070

**Published:** 2016-10-06

**Authors:** Carmen Rueda-Martínez, M. Carmen Fernández, María Teresa Soto-Navarrete, Manuel Jiménez-Navarro, Ana Carmen Durán, Borja Fernández

**Affiliations:** 1 UGC del Corazón, Hospital Universitario Virgen de la Victoria, Universidad de Málaga, Red de Investigación Cardiovascular (RIC), Málaga, Spain; 2 Departamento de Biología Animal, Facultad de Ciencias, Universidad de Málaga, Málaga, Spain; 3 Departamento de Medicina, Facultad de Medicina, Universidad de Málaga, Málaga, Spain; 4 Instituto de Investigación Biomédica de Málaga (IBIMA), Málaga, Spain; Harvard Medical School, UNITED STATES

## Abstract

Bicuspid aortic valve (BAV) is the most frequent congenital cardiac malformation in humans, and appears frequently associated with dilatation of the ascending aorta. This association is likely the result of a common aetiology. Currently, a Syrian hamster strain with a relatively high (∼40%) incidence of BAV constitutes the only spontaneous animal model of BAV disease. The characterization of molecular alterations in the aorta of hamsters with BAV may serve to identify pathophysiological mechanisms and molecular markers of disease in humans. In this report, we evaluate the expression of ten candidate reference genes in aortic tissue of hamsters in order to identify housekeeping genes for normalization using quantitative real time PCR (RT-qPCR) assays. A total of 51 adult (180–240 days old) and 56 old (300–440 days old) animals were used. They belonged to a control strain of hamsters with normal, tricuspid aortic valve (TAV; n = 30), or to the affected strain of hamsters with TAV (n = 45) or BAV (n = 32). The expression stability of the candidate reference genes was determined by RT-qPCR using three statistical algorithms, GeNorm, NormFinder and Bestkeeper. The expression analyses showed that the most stable reference genes for the three algorithms employed were *Cdkn1β*, *G3pdh* and *Polr2a*. We propose the use of *Cdkn1β*, or both *Cdkn1β* and *G3pdh* as reference genes for mRNA expression analyses in Syrian hamster aorta.

## Introduction

Bicuspid aortic valve (BAV) is the most frequent congenital cardiac malformation in humans, with anestimated prevalence of 0.5% - 2% in the general population [1, 2]. BAV is frequently associated with dilatation of the ascending aorta [3, 4]. Affected patients are at risk of aortic dissection or rupture with fatal consequences [5, 6]. Several lines of evidence indicate that the association between BAV and aortic dilatation may derive from a common aetiology. Patients with BAV present a structurally abnormal ascending aorta that predisposes to the aortopathy [4, 6–8]. Although several genetic pathways have been shown to be involved in BAV formation and pathogenesis [4, 7–12], the precise molecular mechanisms leading to BAV and aortic dilatation remain unknown.

Currently, only one spontaneous animal model of BAV disease has been described. It consists of a strain of Syrian hamsters (*Mesocricetus auratus*), with a relatively high (∼40%) incidence of BAV in the general population, not associated with other congenital cardiac malformations [13–15]. Although hamsters with BAV do not develop conspicuous dilatations of the aorta, we have found that these animals show structural alterations of the ascending aortic wall [16].

This study constitutes the first step of a research strategy designed to characterize molecular alterations in the aorta of hamsters with BAV. This strategy may serve to identify pathophysiological mechanisms and molecular markers of disease predisposition in humans. The strategy consists in the identification of differentially expressed genes in the aorta of affected *vs*. normal hamsters, followed by the assessment of their differential expression in the aorta of affected *vs*. healthy human subjects. To do this, quantitative real time PCR (RT-qPCR) is a precise and reliable method [17]. A key aspect of the method is the normalization of mRNA concentrations with housekeeping or reference genes [18, 19], the stability of which must be confirmed in the tissue employed in order to obtain accurate data [18, 20, 21]. However, several common reference genes are frequently used in RT-qPCR assays without further validation, even though they have been found unstable in different tissues and physiological conditions [19, 22– 24], including the dilated ascending aorta [25].

In the present study we tested the stability of ten candidate reference genes in the ascending aorta of hamsters belonging to the affected strain and in control hamsters. Three of these candidate reference genes were identified as the most stable ones in our previous study using human aortic tissue: *Cdkn1β*, *Polr2a* and *Casc3* [26]. The remaining seven reference genes were used by others in studies involving Syrian hamster tissues: *G3pdh*, β-*Actin*, γ*-Actin*, *Pecam-1*, *Rpl18*, *Hprt*, and β*2m* [27, 28].

The selection of the most stable reference genes in our sample population was performed by means of three statistical algorithms, i.e. GeNorm, NormFinder and Bestkeeper [29–31]. These are considered gold standard methods for the selection of appropriate reference genes in gene expression experiments involving RT-qPCR [22, 24, 32–39].

## Material and Methods

### Animals and tissue collection

The animals belonged to two Syrian hamster populations: one inbred (T) and one outbred (H) strain. The T strain shows an elevated incidence (∼40%) of BAV, resulting from a systematic selective inbreeding by mating affected siblings. It was generated in the Department of Animal Biology and maintained in the Animal Facilities of the University of Málaga. The characteristics of this unique strain have been published elsewhere [13–15, 40]. The H strain was used as control. It derives from a closed colony of hamsters that has been outbred since 1990 and commercialized by Janvier (France) (code RjHam: AURA).

The animals were handled in accordance with European and Spanish guidelines for animal welfare, and with the recommendations in the Guide for the Care and Use of Laboratory Animals of the National Institutes of Health. The protocol was approved by the Ethics Committee of Animal Experiments of the University of Málaga (CEUMA; Ethics authorization number: 2015–0006). The animals were housed in standard cages, fed water and chow ad libitum, and sacrificed by CO_2_ inhalation. After death, the chest was opened and the heart, together with the ascending aorta and pulmonary trunk, was dissected out. The ascending aorta, from the sinutubular junction to the branching of the brachiocephalic artery, was excised, immediately immersed in liquid nitrogen, and stored at -80°C for subsequent RNA extraction. The aortic valve was exposed by dissection under the binocular microscope and its morphology assessed. Selected specimens were subsequently analyzed by scanning electron microscopy as previously described [13], in order to document the morphological findings. Briefly, each specimen was fixed by immersion in 1% paraformaldehyde and 2% glutaraldehyde in 0.005 M sodium cacodylate buffer (pH 7.3) for several hours, rinsed with the same buffer, dehydrated with increasing concentrations of ethanol, dried by the critical point method, and gold sputter coated. Observations were made using a Jeol JSM-840 scanning electron microscope.

A total of 107 animals were used, 30 from the H strain (13 males and 17 females; weight: 130–204 g) and 77 from the T strain (33 males and 44 females; weight: 82–126 g). Due to the small size of the ascending aorta of hamsters, and in order to perform an accurate mRNA extraction, two or three aortic specimens were included in each sample. Thus, a total of 12 samples from the H strain and 26 samples from the T strain were obtained. The samples were divided into three groups according to the strain and the aortic valve morphology: samples from animals of the H strain with normal (tricuspid) aortic valve (H-TAV; n = 12, including 30 aortas); samples from animals of the T strain with TAV (T-TAV; n = 15, including 45 aortas); and samples from animals of the T strain with BAV (T-BAV; n = 11, including 32 aortas). According to the age of the animals, the samples were divided into two groups: samples from adult hamsters (n = 17, including 51 aortas from 180–240 days animals) and samples from old hamsters (n = 21, including 56 aortas from 300–440 days old animals).

### RNA Isolation

Each sample was homogenized using IKA ultra-turrax T10B basic homogenizer (LABOTAQ, Spain). Total RNA was extracted using the RneasyMinikit (Quiagen, Germany) following the manufacturer’s instructions. Proteinase K (Sigma-Aldrich, USA) and DNasa I (Quiagen, Germany) were added in order to digest proteins and eliminate any trace of genomic DNA. RNA concentration and purity were evaluated with a Nanodrop D-1000 spectrophotometer (Nanodrop technologies, USA). Only samples with an OD260/280 ratio from 1.8 to 2.1 were selected for further experiments.

### cDNA synthesis

RNA samples were reverse transcribed to cDNA using the High Capacity cDNA Reverse Transcription Kit (Applied Biosystem, USA) according to the manufacturer’s recommendations. The reverse transcriptase reaction was performed with 200 ng RNA in a final volume of 20 μl. The thermocycler conditions consisted of a first step at 25°C for 10 min, followed by a second step at 37°C for 120 min. The samples were then heated at 85°C for 5 min and cooled to 4°C. The cDNA was stored at −20°C until further analysis.

### Real time quantitative PCR (qPCR)

The cDNA was analysed by quantitative real time PCR (qPCR), using the FastStart Universal SYBR Green Master (ROX) (Roche, USA)in an ABI PRISM7500 FastReal-Time PCR Instrument (Applied Biosystem, USA) following the manufacturer’s instructions. An amount of 20ng of cDNAand 300 nM of primer were used for each reaction in a total reaction volume of 20 μl per well.

We analyzed the expression of ten candidate reference genes. The sequence primers for β*-2- microglobulin* (β*2m*), *Hypoxanthine phosphoribosyltransferase* (*Hprt*), *ribosomal protein L18* (*Rpl18*), β*-Actin*, γ*-Actin* and *platelet/endothelial cell adhesion molecule 1* (*Pecam-1*) were obtained from previously published papers [27, 28]. Sequence primers for *glycerol-3-phosphate dehydrogenase* (*G3pdh*) (accession #U10983.1), *Cyclin-dependent Kinase inhibitor 1β* (*Cdkn1β*) (accession # XM_005072872.1), *Polymerase RNA II polypeptide A* (*Polr2a*) (accession # XM_005067838.1), and *Cancer susceptibility candidate 3* (*Casc3*) (accession # XM_005076037.1) were designed by Primer- Blast [41] (Table 1).

**Table 1:** Forward and reverse sequences of the primers employed.

Gene Symbol	Forward sequences	Reverse sequences
***β2m***	GGCTCACAGGGAGTTTGTAC	TGGGCTCCTTCAGAGTTATG
***Hprt***	TGCGGATGATATCTCAACTTTAACTG	AAAGGAAAGCAAAGTTTGTATTGTCA
***Rpl18***	GTTTATGAGTCGCACTAACCG	TGTTCTCTCGGCCAGGAA
***β-Actin***	ACTGCCGCATCCTCTTCCT	TCGTTGCCAATGGTGATGAC
***γ-Actin***	ACAGAGAGAAGATGACGCAGATAATG	GCCTGAATGGCCACGTACA
***Pecam-1***	CAG GAT CAG AAC TTC AGC AAG AT	GCA GCT GAT GGT TAT AGC ATG T
***G3pdh***	GACATCAAGAAGGTGGTGAAGCA	CATCAAAGGTGGAAGAGTGGGA
***Cdkn1β***	CAG CTT GCC GGA GTT CTA CT	ATG CCG GTC CTC AGA GTT TG
***Polr2a***	CTG TGG TGA TGC AGG GTT	CTC ATT CCG CCG TAG CTG AT
***Casc3***	TGTTCTCTCGGCCAGGAA	GGA GAC ATG GAC ACT GGT GG

The cycling conditions were two holding stages, the first at 50°C for 20 sec and the second at 95°C for 10 min, followed by 40 cycles at 95°C for 15 sec andat55°C (*γ-Actin*, *Pecam-1*, *β2m* and *Rpl18*), at 57°C (*β-Actin*, *Cdkn1β*, *G3pdh*, *Hprt* and *Polr2a*) or at 60°C (*Casc3*) for 30 sec, and a Melt Curve stage at 95°C for 15 sec, 60°C for 1 min, 95°C for 30 sec and 60°C for 15 sec. The Melt Curve analyses were performed to confirm the identity and purity of the amplified products. Each reaction was performed twice in order to ensure technical reproducibly of the assays. Each pair of primers was tested in independent plates together with all the samples. A negative control (no template control) was included in each plate in order to test for general contamination. In the ABI 7500 software, the threshold was manually adjusted at the beginning of the exponential phase of amplification. Baseline was automatically calculated by the software.

The comparative delta-CT method was used to normalize gene expression. The LinRegPCR software version 2012.x was used to calculate the amplification efficiency (E) and coefficient of correlation (R^2^) of each primer [42–44]. This software determines PCR efficiency from the slopes of the exponential phase of the individual amplification curves (E = 10^Slope^) and calculates the amplification mean of each amplicon. The correlation coefficient is indicative of the noise in the subset of data points used in the exponential phase to establish the PCR amplification efficiency (32, 44).

### Data analysis

The comparative analysis of the stability of the candidate reference genes was performed with the aid of three different algorithms: GeNorm [29, 33], NormFinder [30, 34] and Bestkeeper [31, 35]. For GeNorm and NormFinder,the mean of the two Ct values obtained from the duplicated PCR reactions was transformed into quantitative data (Q) using the delta-Ct method: Q = E^ΔCt^, where E = amplification efficiency of each amplicon, and ΔCt = lowest Ct value—sample Ct value. Direct average of duplicates Ct values and E values were used for Bestkeeper.

GeNorm software is a Visual Basic application tool that provides the most stable reference gene based on two parameters, the expression stability (M value)and the pairwise variation (V value) [29, 33]. The gene with the lowest M value is considered to have the most stable expression. V value is a guide to determine the optimal number of candidates reference genes required for normalization. When V value is below or equal to 0.15, it is not necessary to include additional reference gene.

Similar to the GeNorm algorithm, NormFinder identifies the gene with the highest expression stability using the M value [30, 34]. However, NormFinder takes into account intragroup and intergroup variation in stability, ranking the best reference genes for normalization.

The Bestkeeper algorithm is also a tool to obtain the most stable reference genes, in this case basing on the analysis of the correlation coefficient of all possible pairs of candidates reference genes [31, 35]. The most stable reference genes are those exhibiting the lowest standard deviation (Std dev) and coefficient of variation (CV) with the highest correlation coefficient (R^2^). Genes that show Std dev greater than 1 are unacceptable.

## Results

### Aortic valve morphology

**Fig 1:**
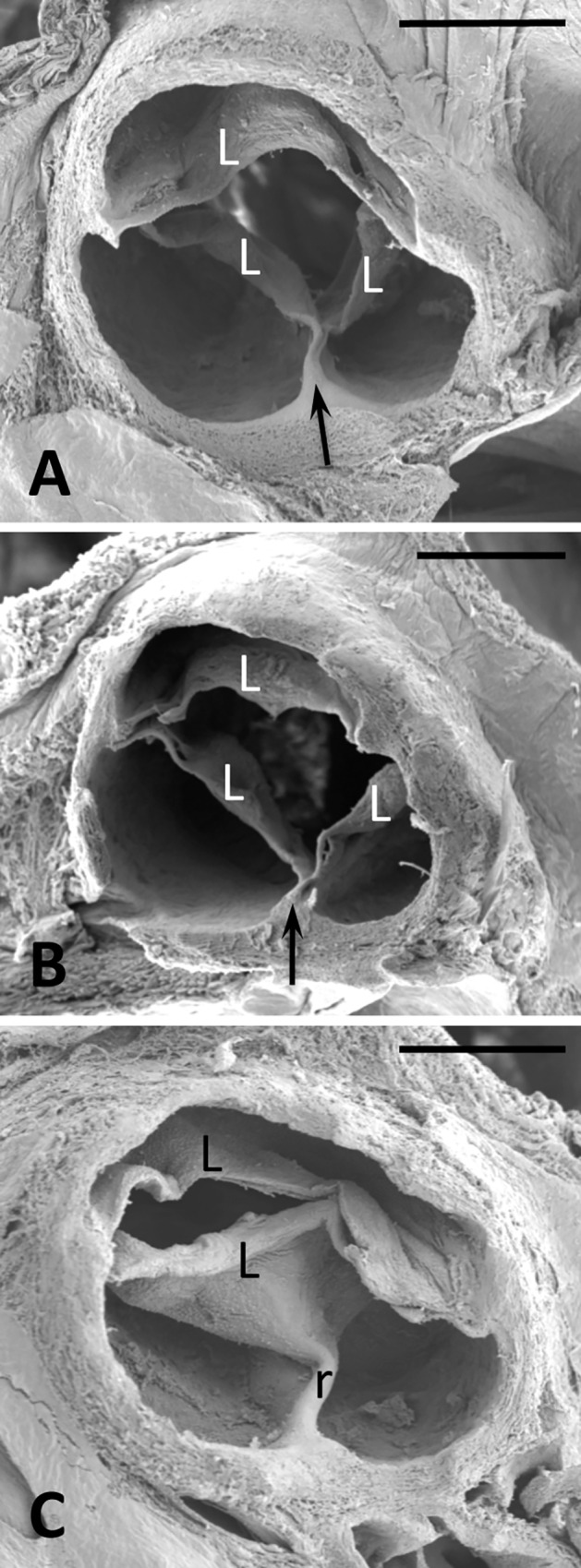
Scanning electron micrographof TAVs (A, B) and a BAV (C) of hamsters from the H (A) and T (B, C) strains. Cranial views. Arrow: commissure; L: leaflet; r: raphe. Bar = 500 μm.

The Fig 1 shows the morphology of representative TAVs and BAVs from hamsters of the H and T strains. TAVs presented three leaflets, three sinuses, and three commissures, whereas BAVs presented two of each of these anatomical elements. All the BAVs showed a dorso-ventral orientation of leaflets and sinuses, thus corresponding to human BAVs type A (A-P).In most BAVs, a raphe was localized in the middle of the ventral aortic sinus, sometimes encroaching towards the ventral leaflet (Fig 1C).

All the hamsters of the H strain (n = 30) showed a TAV. Roughly 60% of hamsters of the T strain showed TAV, whereas the remaining 40% had a BAV. For the present study, we selected 45 animals with TAV and 32 with BAV from the T strain.

### Expression profiles of candidate reference genes

**Fig 2:**
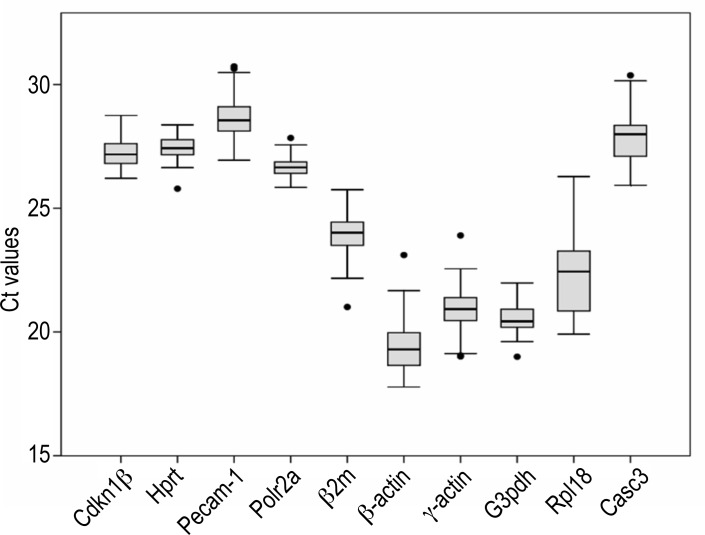
Expression levels of the ten candidate reference genes. Box plot graphs of Ct values for each reference gene tested. Ct values are inversely proportional to the amount of template.

**Fig 3:**
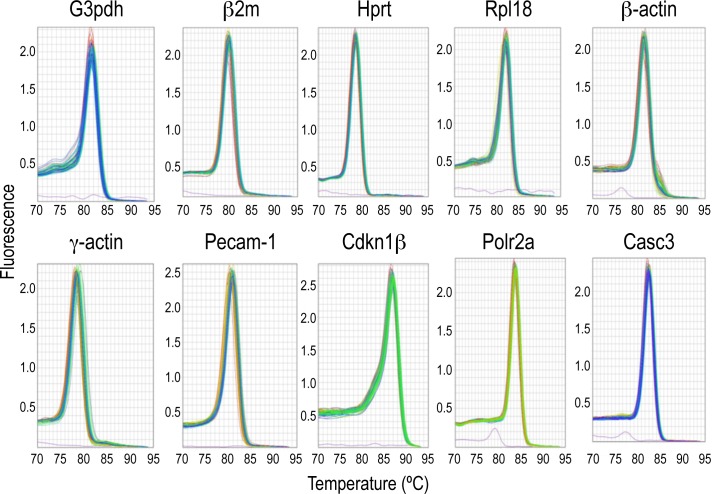
Melting curves of the ten candidate references genes. A single peak represents a specific PCR product.

**Fig 4:**
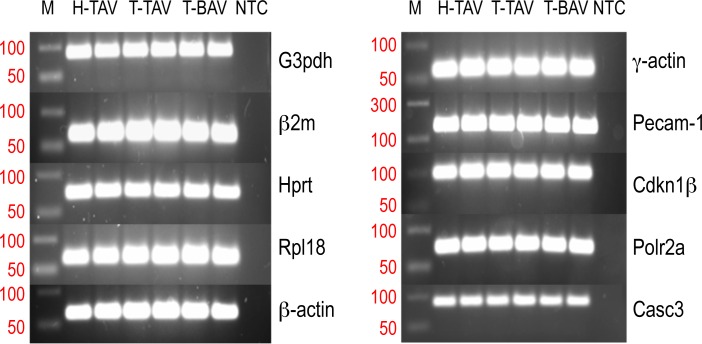
Agarose gel electrophoresis of the PCR products corresponding to the ten candidate reference genes. H-TAV: hamsters with normal valves from the control strain; T-BAV and T-TAV: hamsters with bicuspid or normal valves from the affected strain respectively; M: size markers; NTC: no template control.

The expression levels of the ten candidate reference genes were determined in a total of 38 samples including 107 ascending aortas of hamsters. The average of the duplicated raw Ct values of each amplicon was used to calculate the mean Ct of each gene in the samples (Fig 2). The mean Ct values ranged between 19 and 28. The melting curves indicated a high specificity of the primer set for each amplicon analyzed (Fig 3). One band of the expected size for each amplicon was obtained in a 2% agarosa gel, and no band was detected in the no template control (Fig 4).

The Table 2 shows the amplicon length, the amplification efficiency (E) and the coefficient of correlation (R^2^) of each candidate reference gene. R^2^ was equall to 1 for all genes, indicating a high linearity of all curves, whereas E ranged between 1.7 (85%) and 1.9 (95%) denoting a correct amplification of all amplicons.

**Table 2:** Amplicon length in base pairs (A), correlation coefficient (R^2^)and amplification efficiency (E) of each candidate reference gene.

Gene Symbol	Name	A	R^2^	E (%)
***β2m***	beta 2 microglobulin	77	1	90
***Hprt***	hypoxanthinephosphoribosyltransferase	94	1	90
***Rpl18***	ribosomal protein L18	80	1	85
***β-Actin***	beta- actin	74	1	90
***γ-Actin***	gama-actin	70	1	85
***Pecam-1***	platelet endothelial cell adhesion molecule	192	1	90
***G3pdh***	glyceraldehyde-3-phosphate dehydrogenase	121	1	90
***Cdkn1β***	cyclin-dependent kinase inhibitor 1B	142	1	85
***Polr2a***	polymerase (RNA) II polypeptide A	95	1	90
***Casc3***	cancer susceptibility candidate 3	122	1	95

#### GeNorm analysis

**Fig 5:**
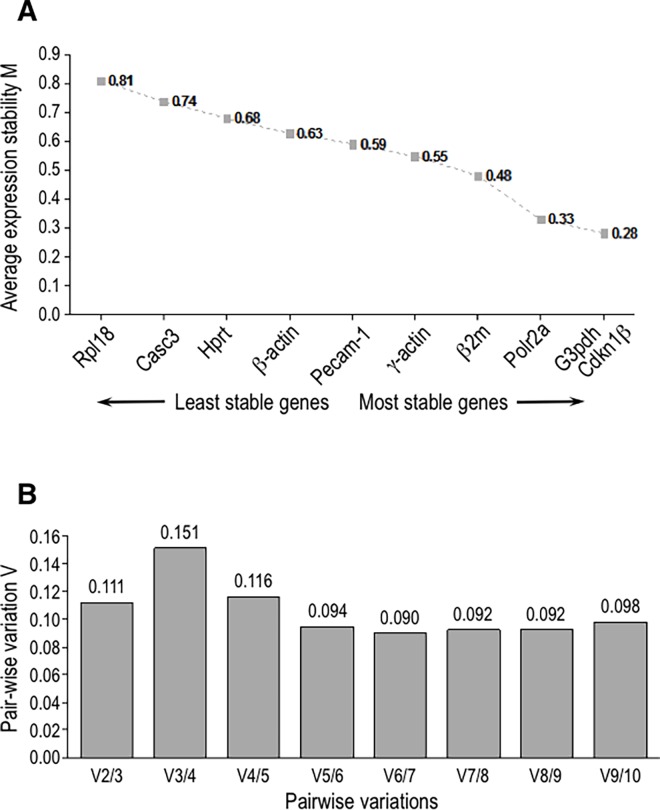
Gene expression stability values (A) and pairwise variation values (B) of the ten candidate reference genes according to the GeNorm algorithm. In A, low M values denote high stability. In B, a V2/3 value below 0.15 indicates that two genes are enough for normalization.

According to the GeNorm analysis, the ten candidate reference genes showed stability (M) values between 0.28 and 0.81 (Fig 5A). The genes *Rpl18* and *Casc3* showed the highest (0.81 and 0.74, respectively) M values (low stability).*Hprt*, *β-Actin*, *Pecam-1*, *γ-Actin* and *β2m* showed intermediate (0.68–0.48) M values. *Polr2a* (M = 0.33), *G3pdh* (M = 0.28) and *Cdkn1β* (M = 0.28) showed the lowest M values, thus representing the most stable reference genes. The pair-wise variation (V) value of 0.11 was obtained for V_2/3_ (Fig 5B), indicating that the two reference genes with the lowest M values are required for accurate normalization.

#### NormFinder Analysis

**Fig 6:**
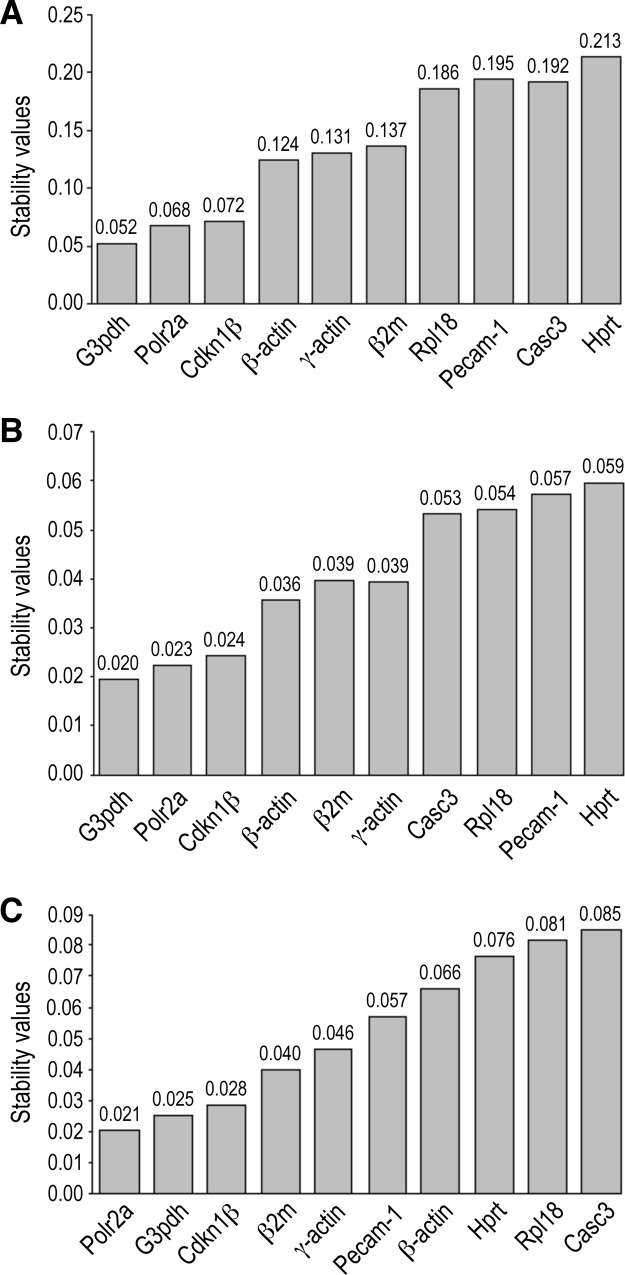
Expression stability values (M) of candidate references genes using the NormFinder software. The M values of the ten genes were calculated using either all the samples in a single group (A), three groups according to the strain and the valve phenotype (B), or two groups of samples according to the age of the animals: adult and old (C).

**Fig 7:**
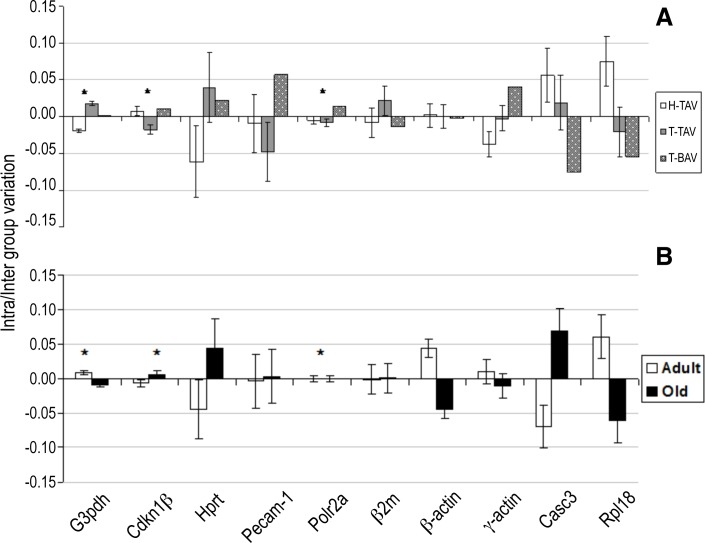
Group variability of candidate references genes using the NormFinder software. Intra-group (error bars) and inter-group (bars) variations of the ten candidate reference genes, according to the strain and aortic valve phenotype (A) or to the age of the animals (B). H-TAV: hamsters with normal valves from the control strain; T-BAV and T-TAV: hamsters with bicuspid or normal valves from the affected strain respectively.

The NormFinder algorithm determined that the most stable reference gene, when all the samples were computed together, was *G3pdh* (M = 0.052) followed by *Polr2a* (M = 0.068) and *Cdkn1β* (M = 0.072) (Fig 6A). When considering three groups of samples (H-TAV, T-TAV and T-BAV), the same ranking was obtained: *G3pdh* (M = 0.020), *Polr2a* (M = 0.023) and *Cdkn1β* (M = 0.024) (Fig 6B). These three reference genes also showed the best combination of intra- and inter-group variation (Fig 7A). When considering two groups of age, the same three genes presented the lowest M values, though with a different ranking: *Polr2a* (0.021), *G3pdh* (0.025) and *Cdkn1β* (0.028) (Fig 6C). These three reference genes also showed the best combination of intra/inter- group variation (Fig 7B).

#### Bestkeeper analysis

According to the Bestkeeper software, the gene Rpl18 exhibited a Std dev value above 1, below the level of acceptance of the test (Table 3). The nine remaining genes showed Std dev below 1. The reference genes *Cdkn1β*, *Polr2a* and *G3pdh* exhibited the best combination of standard deviation, coefficient of variation, and coefficient of correlation, with a p value of 0.001. Thus, the Bestkeeper algorithm indicated that *Cdkn1β*, *Polr2a* and *G3pdh* are the most stable references genes (Table 3).

**Table 3:** Descriptive statistics of the ten candidate reference genes based on the Bestkeeper algorithm.

Genes	GM	Min	Max	Std dev	CV	Std dev (xfold)	R^2^	P-value
***β2m***	23.95	21.01	25.75	0.65	2.69	1.46	0.771	0.001
***Hprt***	27.42	25.79	28.37	0.37	1.36	1.25	0.056	0.736
***Rpl18***	22.21	19.91	26.28	1.27	5.75	2.11	0.758	0.001
***β-Actin***	19.46	17.78	23.11	0.83	4.28	1.63	0.822	0.001
***γ-Actin***	20.96	19.01	24.34	0.80	3.82	1.60	0.858	0.001
***Pecam-1***	28.64	26.94	30.73	0.76	2.64	1.56	0.718	0.001
***G3pdh***	20.49	19	21.98	0.47	2.27	1.32	0.860	0,001
***Cdkn1β***	27.24	26.21	28.75	0.41	1.52	1.27	0.800	0.001
***Polr2a***	26.73	25.84	28.58	0.41	1.53	1.27	0.887	0.001
***Casc3***	27.87	25.93	30.37	0.77	2.76	1.57	0.601	0.001

CV: coefficient of variation; GM: geometric mean of Cp values; Min, Max: minimum and maximum values of Cp; R^2^: coefficient of correlation; Std dev: standard deviation.

## Discussion

The identification of differentially expressed genes in pathological tissues is an invaluable strategy to discover disease specific biomarkers and molecular pathways involved in pathology progression or predisposition. RT-qPCR is the gold standard method to quantify and compare the amount of transcripts in normal and altered tissues [17]. Normalization with reference or housekeeping genes is the most accepted method to allow the comparison of transcript concentrations among samples [18, 19].Several classic reference genes (e.g. *β-Actin*, *G3pdh*, *β2m*) are commonly used with this purpose. However, numerous studies have reported that the expression levels of classical reference genes are not stable in many tissues under certain physiological conditions, making them inadequate for gene expression normalization [19, 22–25]. Accordingly, it is recognized that, in order to obtain reliable data using RT-qPCR analyses, the stability of the housekeeping gene chosen for the experimental setup must be previously demonstrated.

Dilatation of the ascending aorta in patients with BAV is a prevalent aortopathy, the aetiology of which is poorly understood. Numerous studies, aimed to identify molecular mechanisms of disease and biomarkers, have reported differentially expressed genes in dilated aortas of patients with BAV compared to individuals with TAV. However, most of these studies used classical housekeeping genes for normalization, without further validation [45–54].

Currently, the inbred strain of Syrian hamster used in this study (T strain) is the only spontaneous animal model of BAV disease [13–15]. Although hamsters of the T strain do not present aortic aneurysms, they show histological alterations of the ascending aorta, including defective lamellar organization and high incidence of smooth muscle cell death [16]. Given that similar alterations have been described in the aorta of patients with BAV [55, 56], we postulate that the T strain is an appropriate model to study the predisposition to aortopathy associated with BAV. On this basis, we started a research program in order to identify differentially expressed genes in the ascending aorta of hamsters with BAV *vs*. TAV. As a starting point, we have performed the present study, aimed to identify appropriate housekeeping genes for mRNA normalization, which show stable expression in the hamster aorta. We have tested the expression stability of ten candidate reference genes, seven of which (*G3pdh*, *β-Actin*, *γ-Actin*, *Pecam-1*, *Rpl18*, *Hprt* and *β2m*) were previously used as housekeeping genes in hamster tissue [27, 28], and three (*Cdkn1β*, *Polr2a* and *Casc3*) were identified as the most stable reference genes in human ascending aorta [26].The comparison of the expression stability of the different reference genes in the three groups of hamsters have been performed with the aid of the GeNorm, NormFinder and Bestkeeper algorithms [29–31,33–35], which are considered gold standard methods for this purpose [22, 24, 32–39].

All the candidate references genes showed raw Ct values ranging from 19 to 28 (Fig 2), melting curves and PCR bands consistent with specific amplifications (Figs 3 and 4), high amplification efficiencies, and maximal correlation coefficient (Table 2). These data indicated that the ten candidate references genes were suitable for subsequent analysis of stability.

The results of GeNorm analysis showed that the most stable reference genes in the samples studied are *Cdkn1β* and *G3pdh* (M = 0.28), followed by *Polr2a* (M = 0.33) (Fig 5A). These M values were rather lower than those of the other candidate housekeepings. In addition, the pair-wise variation analysis employed by GeNorm suggested that two reference genes, in this case *Cdkn1β* and *G3pdh*, must be used for normalization (Fig 5B). It should be noted, however, that GeNorm considers a minimum of two housekeepings for normalization. NormFinder algorithm takes into account intra- and inter-groups variations when studying heterogeneous populations. When NormFinder calculated the stability value considering a single sample population, the ranking of the most stable housekeeping genes was *G3pdh*, *Polr2a* and *Cdkn1β* (Fig 6A). When NormFinder considered three groups of specimens (H-TAV, T-TAV and T-BAV), the same ranking was obtained (*G3pdh*, *Polr2a* and *Cdkn1β*) (Fig 6B). However, when considering two groups of specimens (adult and old animals), the ranking of the most stable housekeeping genes varied (*Polr2a*, *G3pdh* and *Cdkn1β*) (Fig 6C). According to the values yielded by the algorithm NormFinder, these three genes had the lowest intra- and inter-group variation (Fig 7). Finally, the Bestkeeper algorithm identified three reference genes (*Cdkn1β*, *Polr2a* and *G3pdh*) with the best combination of standard deviation (lowest), coefficient of variation(lowest) and coefficient of correlation (highest) (Table 3).

In summary, all the three algorithms (NormFinder, GeNorm and Bestkeeper) employed to compare the expression stability of our ten candidate reference genes agreed in the identification of *Cdkn1β*, *G3pdh*and *Polr2a* as the most stable reference genes in our sample population (Table 4). However, the three algorithms resulted in slightly different rankings of stability. This variation probably denotes differences in input data and mathematical models employed by the softwares [26, 36, 57].

**Table 4:** Ranking of the quality of the reference genes according to GeNorm, NormFinder and Bestkeeper algorithms.

Ranking	GeNorm	NormFinder	Bestkeeper
**1**	*G3pdh/Cdkn1β*	*G3pdh/Cdkn1β*	*Cdkn1β/Polr2a*
**2**	*Polr2a*	*Polr2a*	*G3pdh*
**3**	*β2m*	*β-Actin*	*β2m*
**4**	*γ-Actin*	*β2m/ γ-Actin*	*Pecam-1*
**5**	*Pecam-1*	*Casc3*	*Casc3*
**6**	*β-Actin*	*Rpl18*	*γ-Actin*
**7**	*Hprt*	*Pecam-1*	*β-Actin*
**8**	*Casc3*	*Hprt*	*Hprt*
**9**	*Rpl18*		*Rpl18*

Although most studies involving RT-qPCR analyses use a single housekeeping gene for normalization, it has been suggested that this approach leads to appreciable errors. Thus, the usage of the geometric mean of multiple carefully selected housekeeping genes has been proposed as the most accurate normalization factor for RT-qPCR experiments [33]. In the present study, *Cdkn1*β and *G3pdh* were identified by GeNorm as the reference genes to be used for proper normalization of transcripts in aortic tissue of Syrian hamsters. Nevertheless, we have recently performed RT-qPCR analyses of human ascending aorta from patients with normal aorta, dilated aorta, BAV, and TAV, and we have obtained similar results when using one (*Cdkn1β*) or three (*Cdkn1β*, *Polr2a* and *Casc3*) validated reference genes to normalize gene expression [58].

Interestingly, using the same methodological approach as in this work, we identified *Cdkn1β* and *Polr2a* as the two most stable reference genes in normal and disease aortic tissue from human patients with TAV and BAV [26]. The fact that in the present study these two housekeeping genes are ranked as the first and third most stable genes in aortic tissue from hamsters with TAV and BAV, strengthens the hypothesis that the T strain of hamsters is an appropriate animal model to study the molecular mechanisms involved in the development of aortic dilatation associated with BAV in humans.

In conclusion, by analyzing RT-qPCR data with the three gold standard mathematical algorithms for housekeeping gene identification, we have found that *Cdkn1β*, followed by *G3pdh*, are the most stable reference genes in ascending aorta of Syrian hamsters with different aortic valve morphologies and age. Our present results will contribute to identify potentially relevant molecular players and markers in the development and progression of BAV disease.
